# Pathoplastic effects of metabolic disorders in severe mental illness

**DOI:** 10.1192/j.eurpsy.2022.741

**Published:** 2022-09-01

**Authors:** M. Fiorani, L. Orsolini, U. Volpe, V. Salvi

**Affiliations:** Unit of Clinical Psychiatry, Polytechnic University of Marche, Ancona, Italy, Department Of Neurosciences/dimsc, Ancona, Italy

**Keywords:** severe mental illness, metabolic syndrome, Insuline resistance, Pathoplastic effect

## Abstract

**Introduction:**

Patients with severe mental illness (SMI) have a higher risk of weight gain, dyslipidemia and insulin-resistance. It was observed that insulin resistance has a pathoplastic effect: in Schizophrenia it was associated with a greater severity of negative symptoms, whereas in Bipolar Disorder it was associated with more chronicity and rapid cycling. Moreover a correlation was observed between obesity and a worse outcome in Bipolar Disorder type I.

**Objectives:**

We aimed at assessing the influence of dysmetabolisms on clinical characteristics in patients with SMI.

**Methods:**

We recruited 78 patients with SMI consecutively hospitalized in the Psychiatry Clinic of the Ospedali Riuniti of Ancona, Italy. We administered a checklist for socio-demographic and clinical features (diagnosis, age of onset, illness duration, number of episodes, number of episodes per year, suicidal attempts and comorbidities), and evaluated the following metabolic parameters: weight, height, BMI, abdominal circumference, blood pressure, total cholesterol, HDL, triglycerides, glycemia and insulinemia. We determined insulin-resistance according to the HOMA-IR model. We performed bivariate Pearson correlations to compare metabolic and socio-demographic/clinical parameters.

**Results:**

The analyses showed positive correlations between BMI and disease duration (P = 0.003), and BMI and the number of episodes (P = 0.022). Furthermore, a positive correlation was found between HOMA-IR and the number of episodes per year (P = 0.008). The associations remained statistically significant after controlling for age through partial correlations.

**Conclusions:**

Weight gain and insulin-resistance in severe mental illness are associated with a more severe SMI, as suggested by the greater number of acute episodes.

**Disclosure:**

No significant relationships.

